# Synthetic Lethal Interactions between EGFR and PARP Inhibition in Human Triple Negative Breast Cancer Cells

**DOI:** 10.1371/journal.pone.0046614

**Published:** 2012-10-11

**Authors:** Somaira Nowsheen, Tiffiny Cooper, Jennifer A. Stanley, Eddy S. Yang

**Affiliations:** 1 Department of Radiation Oncology, Comprehensive Cancer Center, University of Alabama at Birmingham School of Medicine, Birmingham, Alabama, United States of America; 2 Department of Cell, Developmental, and Integrative Biology, University of Alabama at Birmingham School of Medicine, Birmingham, Alabama, United States of America; 3 Department of Pharmacology and Toxicology, Comprehensive Cancer Center, University of Alabama at Birmingham School of Medicine, Birmingham, Alabama, United States of America; Wayne State University School of Medicine, United States of America

## Abstract

Few therapeutic options exist for the highly aggressive triple negative breast cancers (TNBCs). In this study, we report that a contextual synthetic lethality can be achieved both *in vitro* and *in vivo* with combined EGFR and PARP inhibition with lapatinib and ABT-888, respectively. The mechanism involves a transient DNA double strand break repair deficit induced by lapatinib and subsequent activation of the intrinsic pathway of apoptosis. Further dissection of the mechanism reveals that EGFR and BRCA1 can be found in the same protein complex, which is reduced by lapatinib. Interestingly, lapatinib also increases cytosolic BRCA1 and EGFR, away from their nuclear DNA repair substrates. Taken together, these results reveal a novel regulation of homologous recombination repair involving EGFR and BRCA1 interaction and alteration of subcellular localization. Additionally, a contextual synthetic lethality may exist between combined EGFR and PARP inhibitors.

## Introduction

Breast cancer is a heterogeneous disease comprising various subgroups with unique molecular signatures. One of the subtypes, triple negative breast cancer (TNBC, estrogen receptor negative, progesterone receptor negative, and human epidermal growth factor receptor negative), is an aggressive form of breast cancer with a high potential for metastasis and resistance to standard therapies. The disease lacks a well-defined therapeutic target. Angiogenesis inhibitors, epidermal growth factor receptor (EGFR)-targeted agents, and src kinase and mTOR inhibitors are among the therapeutic agents being actively investigated in clinical trials in patients with TNBC but have, thus far, failed to show promise [1].

PARP inhibitors induce synthetic lethality by targeting homologous recombination (HR)-mediated DNA repair deficient tumors while maintaining minimal normal tissue toxicity [2], [3]. However, this approach is only applicable to the 5–10% of all cancers with hereditary mutations in key proteins in the HR pathway. Thus, much effort has been undertaken to expand the utility of PARP inhibitors beyond the current realms of BRCA-associated tumors by combining with agents that alter the DNA damage/repair pathways. Specifically, in TNBC, which often demonstrates a “BRCAness" phenotype, PARP inhibitors showed initial promise when combined with DNA damaging chemotherapy, but ultimately failed to improve outcomes over chemotherapy alone in a phase III trial [4].

EGFR, a proto-oncogene that belongs to a family of four transmembrane receptor tyrosine kinases that mediate the growth, differentiation, and survival of cells, is often overexpressed in TNBC and is associated with aggressive disease phenotype [1], [5], [6], [7]. However, targeted therapy against EGFR using the anti-EGFR monoclonal antibody cetuximab had limited activity as a single agent in TNBC [8], [9].

We and others have previously shown that EGFR inhibition alters the DNA DSB repair capacity of treated cells [10], [11]. Here we report that lapatinib, a dual EGFR1/2 inhibitor, induces a transient DNA repair deficit in human triple negative breast cancer cells both *in vitro* and *in vivo* and subsequently augments cytotoxicity to the PARP inhibitor ABT-888. The mechanistic insight of this enhanced sensitivity involves lapatinib-induced reduction of nuclear BRCA1 and EGFR, which compromises HR-mediated DNA double strand break repair, generates persistent DNA damage, and subsequently renders sporadic TNBCs susceptible to ABT-888. Our intriguing results reveal a novel regulation of homologous recombination repair involving EGFR and BRCA1 interaction and subcellular localization and suggest that combining EGFR and PARP inhibition results in greatest cytotoxicity compared to either alone.

## Materials and Methods

### Ethics statement

All experiments conducted were approved by the University of Alabama at Birmingham Occupational Health & Safety Board. All animal procedures were approved by the University of Alabama at Birmingham Institutional Animal Care and Use Committee.

### Cell culture

The human breast carcinoma cell line MDA-MB-231 (HTB-26) were obtained from ATCC (Manassas, VA) and cultured in RPMI (Invitrogen) supplemented with 10% fetal bovine serum (FBS, Atlanta Biologicals). MDA-MB-453 (HTB-131, ATCC) and MDA-MB-468 (HTB-132, ATCC) cell lines were obtained courtesy of Dr. Donald Buchsbaum (University of Alabama at Birmingham, Birmingham, AL) and cultured in DMEM (Invitrogen) supplemented with 10% FBS.

### Drugs, plasmids and transfection

ABT-888 was obtained from Enzo Life Sciences (catalog # ALX-270-444) while lapatinib (catalog # L-4804) was obtained from LC Laboratories. DR-GFP to measure chromosomal HR repair capacity, ISce-1 and the empty vector were gifts from Dr. Fen Xia (Ohio State University, OH) and has been described previously [12], [13]. All transfections were performed using Lipofectamine according to the manufacturer's recommendations (Invitrogen).

### Clonogenic survival assay

Cell survival was evaluated by the colony formation assay in the breast cancer cell lines as previously described [10], [14]. Briefly, cells were seeded and treated with the indicated doses of drugs (lapatinib, ABT-888 or vehicle) following which the plates were left undisturbed. Three weeks following treatment, colonies were fixed with 70% ethanol, stained 1% methylene blue (Sigma) and number of positive colonies were counted (>50 cells). Survival fraction was calculated as follows: (number of colonies for treated cells/number of cells plated)/(number of colonies for corresponding control/number of cells plated). Experiments were performed at least in triplicate.

### Apoptosis analysis

Apoptosis was analyzed using the Annexin V-FITC Apoptosis Detection kit (BioVison Research Products; catalog # K101-400) according to manufacturer's instructions and as previously described [14]. Briefly, cells were exposed to vehicle or lapatinib for 16 hours, treated with vehicle or ABT-888 and collected 40 hours post lapatinib treatment for analysis via flow cytometry. Experiments were performed at least in triplicate.

### Cellular fractionation, co-immunoprecipitation and immunoblotting

Immunoblotting was performed as described previously [10], [14], [15]. Briefly, cell lysates were prepared using radioimmunoprecipitation lysis buffer (150 mM NaCl, 50 mM Tris, pH 8.0, 5 mM EDTA, 0.5% sodium deoxycholate, 0.1% SDS, 1.0% Nonidet P-40) supplemented with protease and phosphatase inhibitor cocktails (Sigma) and subjected to SDS-PAGE analysis. Cellular fractionation was performed to assay BRCA1 and EGFR location and co-immunoprecipitation to assay BRCA1 and EGFR interaction following 16 hours exposure to lapatinib as previously described [14]. All antibodies were used at dilutions recommended by the manufacturer: β-Actin (Santa Cruz Biotechnology, catalog # sc-47778), Histone H1 (Santa Cruz Biotechnology, catalog # sc-10806), Tubulin (Santa Cruz Biotechnology, catalog # sc-53646), BRCA1 (Abcam, catalog # OP-92), EGFR (Santa Cruz Biotechnology, catalog # 81449), caspase 3 (Cell Signaling, catalog # 9688), cleaved caspase 3 (Cell Signaling, catalog # 9664), caspase 9 (Cell Signaling, catalog # 9502), and cleaved caspase 9 (Cell Signaling, catalog # 9501).

### Immunofluorescence

To assay HR-mediated DNA double strand break repair in breast cancer cell lines, immunohistochemistry for radiation-induced Rad51 foci was performed as previously described [10], [14]. Briefly, cells were exposed to 0.1 µM-1 µM lapatinib for 16 hours, and subsequently treated with mock or 3 Gy γ-IR using an X-ray irradiator at 1.225 Gy/min (Kimtron Inc., Woodbury, CT). To assay levels of persistent DNA damage as measured via γ-H2AX cells were exposed to lapatinib for 16 hours followed by ABT-888 and fixed at the indicated time points. The following antibodies were utilized: Rad51 (1∶500 dilution, Santa Cruz Biotechnology, catalog # sc-8349), Alexa Fluoro 488 anti-rabbit (1∶2000 dilution, Invitrogen, catalog # A11034), and γ-H2AX Ser139 (1∶500 dilution, Millipore, catalog #07-164).

### Chromosomal homologous recombination mediated repair analysis

MDA-MB-231 cells were transfected with DRGFP substrate and stable integrants were selected with 2 µg/mL of puromycin (Sigma) for 3 weeks. Puromycin-resistant colonies were isolated and expanded. Chromosomal HR-mediated repair capacity was determined as described previously [12], [13]. Breast cancer cell lines stably expressing the DRGFP repair substrate were treated as required and subsequently transfected with either an empty vector, ISce-1 expression vector to measure HR-mediated repair capacity, or a GFP expression vector to measure transfection efficiency. Two days after transfection with ISceI expression plasmid or empty vector, cells were subjected to two-color fluorescence analysis, which revealed the percentage of GFP+ cells relative to the total cell number. For each analysis, 100,000 cells were processed. All transfections were performed using Lipofectamine. HR relative to total transfected cells was determined by division of the % GFP+ cells from each ISce-1 transfection by the % GFP+ cells from a parallel GFP transfection. 7-Aminoactinomycin D (7-AAD, Invitrogen) was used as well to control for cell viability.

### Tumor Growth Delay

NOD.CB17-Prkdcscid/J female mice, age 3 weeks, were obtained from Jackson Laboratories. After a seven-day acclimatization period, 5×10^6^ MDA-MB-231 cells were orthotopically injected into the mammary fat pad. Once the tumors were palpable, the mice were weighed and randomized into four groups (n = 7): control, ABT-888, lapatinib, or ABT-888+lapatinib. Mice were subsequently treated with ABT-888 (100 mg/kg) and/or lapatinib (30 mg/kg) by oral gavage twice daily for 26 days. Tumor volume was measured with digital calipers 3 times per week and calculated using the equation: (width×length×height)/2. All animal procedures were approved by the University of Alabama at Birmingham Institutional Animal Care and Use Committee.

### Statistical analysis

The data were analyzed via analysis of variance (ANOVA) followed by a Bonferroni post test using GraphPad Prism version 4.02 (GraphPad Software, San Diego, CA). Data presented as average +/− standard error of mean.

## Results

### Contextual synthetic lethality with EGFR and PARP inhibition in triple negative breast cancer cells

**Figure 1:**
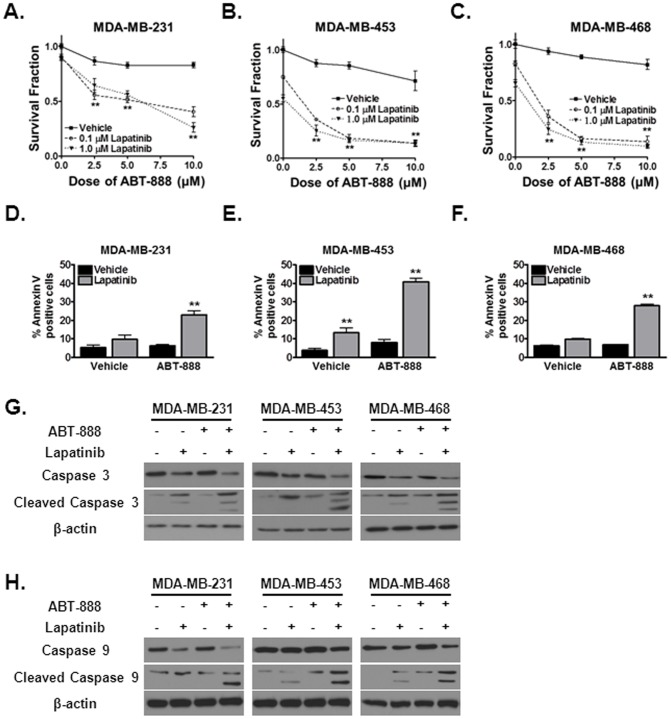
Targeting EGFR pathways using lapatinib augments cytotoxicity to ABT-888 in triple negative breast cancer and promotes intrinsic apoptosis. (A–C) ABT-888 with lapatinib reduces the colony forming ability of human triple negative breast cancer cells. (A) MDA-MB-231, (B) MDA-MB-453 and (C) MDA-MB-468 cell lines were seeded for colony formation assay and treated with 0.1 µM lapatinib, 1 µM lapatinib, or vehicle control. 16 hours following initial treatment, the cells were exposed to different doses of ABT-888 or vehicle control. Shown is the mean survival fraction (+/− SEM) from at least three independent experiments (**p<0.01). (D–F) Lapatinib and ABT-888 increases apoptosis in (D) MDA-MB-231, (E) MDA-MB-453 and (F) MDA-MB-468 cell lines as evidenced by increased percentage of Annexin V positive cells. Cells were subjected to either vehicle or 1 µM lapatinib for 16 hours and subsequently exposed to 10 µM ABT-888. 24 hours following the treatment period cells were subjected to flow cytometry. Shown is the mean % of Annexin V positive cells (+/− SEM) from at least three independent experiments (**p<0.01). (G–H) Lapatinib and ABT-888 increases intrinsic apoptosis in MDA-MB-231, MDA-MB-453 and MDA-MB-468 cell lines as evidenced by cleavage of (G) caspase 3 and (H) caspase 9. Cells were subjected to either vehicle or 1 µM lapatinib for 16 hours and subsequently exposed to 10 µM ABT-888. 24 hours following the treatment period cell lysates were harvested and levels of total and cleaved caspase 3 and 9 were detected by immunoblotting. A dramatic increase in cleaved caspase 3 and 9 with a concurrent reduction in total caspase was observed. Actin was used as a loading control. Shown are representative western blots from at least three independent experiments.

We recently reported that cetuximab, which inhibits the EGFR signaling pathway, can generate a DNA repair defect in head and neck cancer cells and subsequently induce a contextual synthetic lethality with the PARP inhibitor ABT-888 [11]. We thus hypothesized that lapatinib, a dual tyrosine kinase inhibitor which interrupts the HER1/HER2 growth receptor pathways, would generate a similar DNA repair deficit and induce susceptibility to ABT-888 in human TNBC cells [16]. Consistent with our hypothesis, lapatinib in combination with ABT-888 significantly reduced the survival fraction in a dose dependent manner (70–99%) in the well characterized human TNBC cell lines MDA-MB-231 (Figure 1A), MDA-MB-453 (Figure 1B) and MDA-MB-468 (Figure 1C) [17], [18], [19]. Lapatinib alone produced a 10–30% reduction in the survival fraction of these cells. These novel and intriguing results suggested that indeed, the EGFR pathway can be targeted in TNBC cells to render them susceptible to ABT-888.

### Enhanced cytotoxicity with lapatinib and ABT-888 involves activation of the intrinsic apoptotic pathway

Given that as little as one DNA double strand break is lethal to the cell, and since PARP inhibition in DNA repair deficient cells has been shown induce the apoptotic pathway, we next examined activation of cellular apoptosis to further elucidate the mechanism by which lapatinib and ABT-888 induce cellular cytotoxicity. To assay apoptosis, we first analyzed the percentage of Annexin V positive cells, an early indicator of apoptosis induction, following vehicle, lapatinib, ABT-888, or combination treatment. As shown in Figure 1D–F, activation of apoptosis was significantly greater in MDA-MB-231 (Figure 1D), MDA-MB-453 (Figure 1E), and MDA-MB-468 (Figure 1F) cell lines with lapatinib and ABT-888 compared to either agent alone suggesting a synergistic relationship between these therapeutic agents.

Activation of apoptotic pathways ultimately leads to cleavage of caspase 3, which in turn initiates the cascade of proteolysis of integral cellular proteins and results in programmed cell death. To confirm an induction of apoptosis with the combination of lapatinib and ABT-888 in TNBC cells, we next assessed the levels of total and cleaved caspase 3 following PARP and EGFR inhibition. As shown in Figure 1G, increased cleaved caspase 3 with a concomitant reduction in total or uncleaved caspase 3 was observed in all the TNBC cell lines studied. Activation of apoptosis was significantly greater in MDA-MB-231, MDA-MB-453, and MDA-MB-468 cell lines with lapatinib and ABT-888 compared to either agent alone. Consistent with the colony formation assays, lapatinib alone induced apoptosis in treated cells as well but the levels were significantly lower compared to lapatinib+ABT-888.

Cellular apoptosis can be induced via the intrinsic or extrinsic pathways [20]. The extrinsic pathway is activated by proapoptotic ligand-mediated stimulation of cellular death receptors while the intrinsic pathway is triggered by stress signals from within the cell. We hypothesized that the PARP inhibitor induced apoptotic response is, at least in part, due to intracellular stress signals from DNA damage leading to activation of the intrinsic apoptotic pathway. To further dissect the apoptotic pathways activated by combined EGFR/PARP inhibition, we investigated cleavage of caspase 9 in treated cells. As shown in Figure 1H, enhanced cleavage of caspase 9 was observed following lapatinib/ABT-888. These data support activation of the intrinsic apoptotic pathway following lapatinib and ABT-888 treatment.

### Lapatinib induces a homologous recombination-mediated repair deficiency in triple negative breast cancer cells

**Figure 2:**
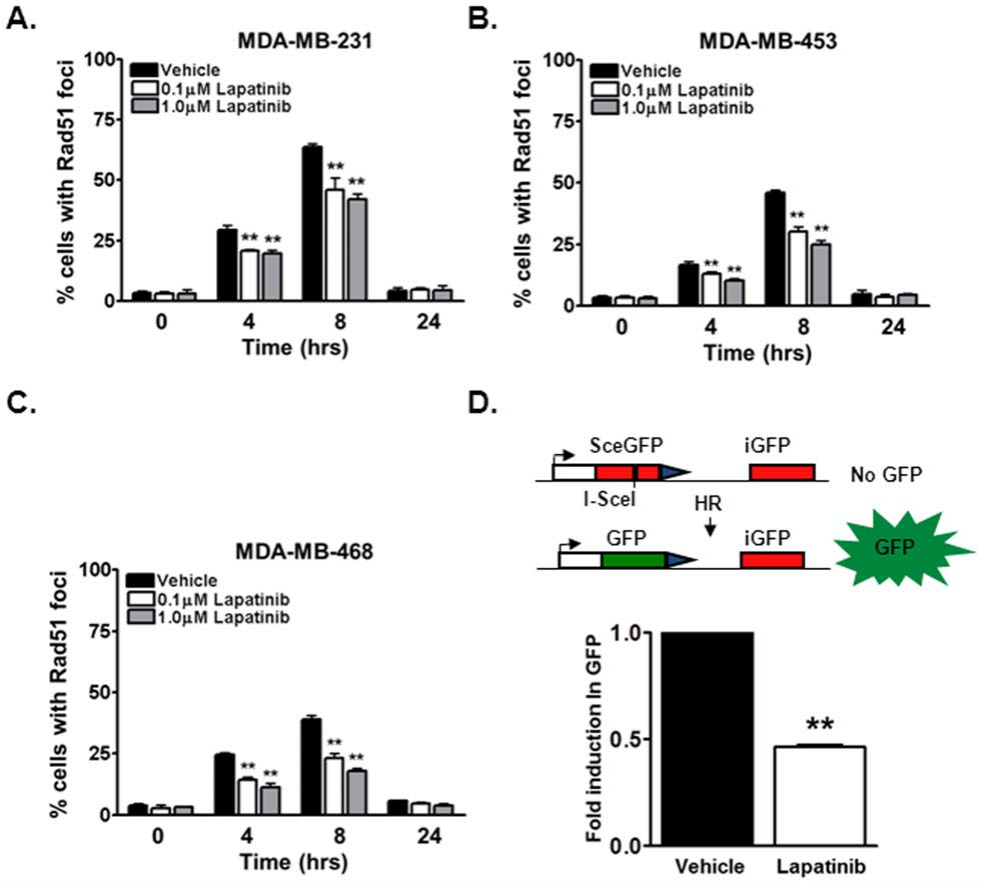
Lapatinib attenuates homologous recombination mediated DNA double strand break repair in triple negative breast cancer cells. (A–C) Homologous recombination (HR) repair capacity was measured in (A) MDA-MB-231, (B) MDA-MB-453 and (C) MDA-MB-468 triple negative breast cancer cell lines by assessing radiation-induced rad51 foci, a well characterized marker for HR repair. Briefly, cells were exposed to mock or 3 Gy irradiation (IR) and subsequently subjected to immunofluorescence staining for rad51 foci. Shown is the representative data of 3 independent experiments the % of cells (mean +/− SEM) with rad51 foci (**p<0.01 compared to vehicle). (D) Chromosomal HR repair capacity was directly measured in MDA-MB-231DRGFP cells. MDA-MB-231DRGFP were treated with 1 µM lapatinib or vehicle control. 16 hours following the treatment period, cells were transfected with ISce-1 or control vector. 48 hours following transfection cells were subjected to flow cytometry for GFP expression. Shown is the representative fold induction in GFP (mean +/− SEM) from at least 3 independent experiments (**p<0.01 compared to vehicle). Inset is a representative figure depicting the DRGFP repair model.

Our data thus far supports a potential contextual synthetic lethal interaction between EGFR and PARP inhibition, which suggests that lapatinib may induce a HR repair deficiency. To assess this notion, we first analyzed radiation-induced rad51 foci, a well-established functional marker of HR repair activity [10], [12], [14], [21]. As shown in Figure 2A–2C, a robust time-dependent induction in rad51 levels was observed in MDA-MB-231 (peak 65% 8 hours following radiation, Figure 2A), MDA-MB-453 (peak 46% 8 hours following radiation, Figure 2B), and MDA-MB-468 (peak 40% 8 hours following radiation, Figure 2C) cells. However, lapatinib significantly attenuated the formation of rad51 foci in MDA-MB-231 (40% 8 hours following lapatinib and radiation, Figure 2A), MDA-MB-453 (25% 8 hours following lapatinib and radiation, Figure 2B), and MDA-MB-468 (17% 8 hours following lapatinib and radiation, Figure 2C) cells.

We also directly measured the effects of lapatinib on HR using a GFP-based chromosomal HR repair assay [12], [13]. In these assays, MDA-MB-231 cell lines stably expressing the DRGFP HR repair substrate were generated (MDA-MB-231DRGFP). These cells were exposed to lapatinib, and the HR-mediated repair of an endonuclease generated DNA double strand break was measured by assessing the % of GFP positive cells, indicative of HR-mediated repair. As shown in Figure 2D, treatment with lapatinib significantly attenuated the percentage of GFP positive cells (2.53% vs 1.16%) by approximately 2 fold. Since reduction in HR-mediated repair may also be due to cell cycle affects, cell cycle distribution was analyzed following lapatinib treatment. No significant redistribution in cell cycle was observed at the time points at which repair was analyzed (Table S1). Thus, these results confirm the notion that lapatinib generates a HR-mediated DNA double strand break repair deficiency independent of cell cycle effects.

### Enhanced cytotoxicity with lapatinib and ABT-888 involves persistent DNA damage

**Figure 3:**
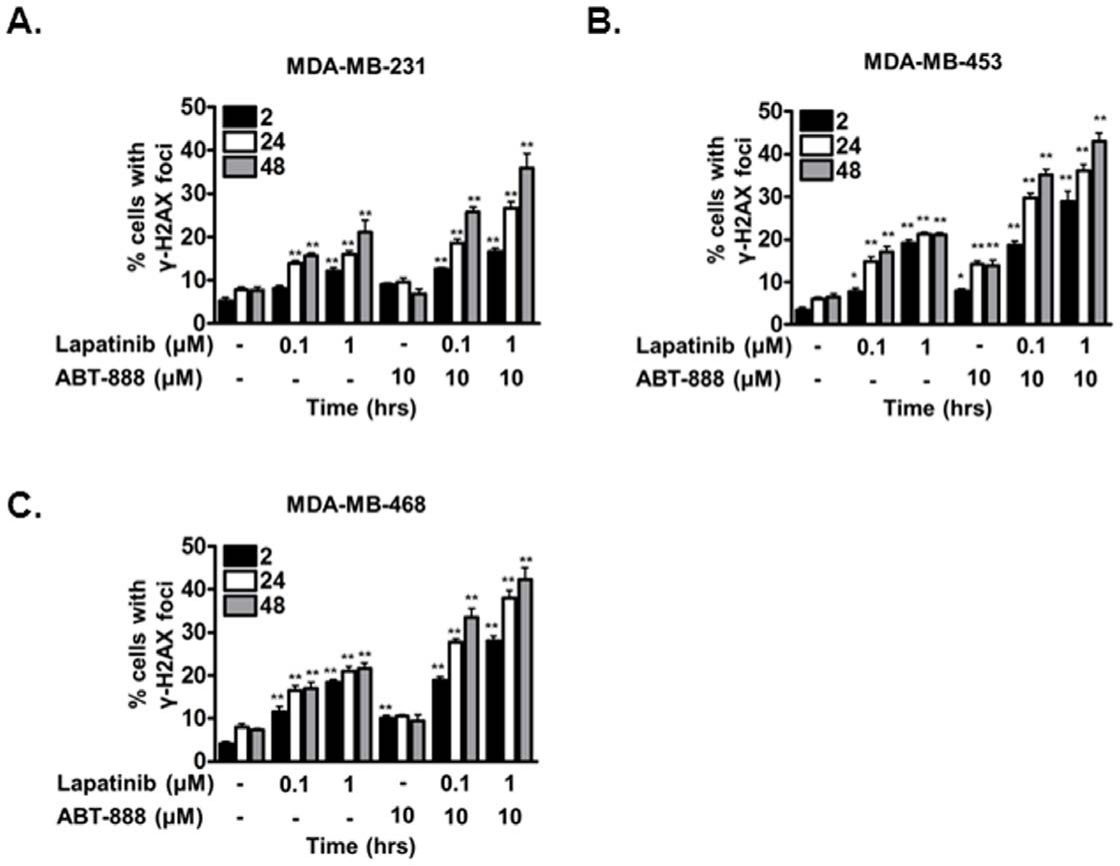
Combination lapatinib and ABT-888 induces persistent DNA double strand break damage in triple negative breast cancer cells. (A–C) DNA damage 2, 24, and 48 hours following vehicle, lapatinib, ABT-888, or both lapatinib+ABT-888 was assessed by γ-H2AX foci in (A) MDA-MB-231, (B) MDA-MB-453 and (C) MDA-MB-468 cell lines. Cells were treated with vehicle or various doses of lapatinib for 16 hours and subsequently exposed to vehicle or 10 µM ABT-888. At the indicated times following PARP inhibition, cells were processed for immunofluorescence staining for γ-H2AX foci. Shown is the representative data of 3 independent experiments the % of cells (mean +/− SEM) with foci (*p<0.05, **p<0.01).

PARP inhibitor inhibits the base excision repair pathway responsible for the resolution of DNA single strand breaks. SSBs that persist in dividing cells are ultimately converted to double strand breaks and repaired by HR-mediated repair. Because EGFR inhibition with lapatinib induced a HR defect, we hypothesized that the enhanced cytotoxicity of TNBC to lapatinib and ABT-888 may be due to persistent DNA double strand breaks. Thus, to assess the levels of DNA damage in the TNBC cell lines, we analyzed γ-H2AX foci, a well-established functional marker of DNA double strand break [22]. As shown in Figure 3, indeed, a robust induction in γ-H2AX levels was observed in MDA-MB-231 (37% following lapatinib+ABT-888 treatment vs 7% in control, Figure 3A), MDA-MB-453 (45% following lapatinib+ABT-888 treatment vs 6% in control, Figure 3B), and MDA-MB-468 (45% following lapatinib+ABT-888 treatment vs 7% in control, Figure 3C) cells. Compared to vehicle control, lapatinib alone as expected induced a 2–3 fold increase in the percentage of cells exhibiting persistent DNA double strand breaks. Interestingly, the combination of lapatinib and ABT-888 resulted in a significantly greater number of cells with persistent DNA damage in all cell lines examined (Figure 3). As expected, ABT-888 alone did not result in significant increase in cells with persistent DNA double strand break damage except a mild increase in MDA-MB-453 (Figure 3B). Thus, the mechanism of enhanced cytotoxicity with lapatinib and ABT-888 involves persistent DNA damage and the cytotoxicity from lapatinib and ABT-888 may be due to the inability of treated cells to resolve DNA DSBs, the most critical lesion in cells.

### Lapatinib induces cytosolic translocation of key DNA repair proteins

**Figure 4:**
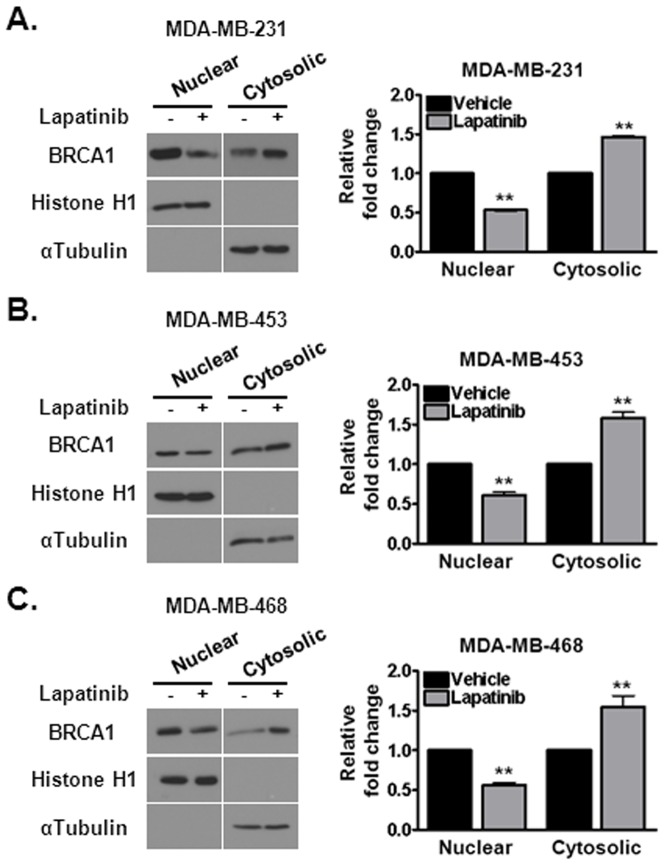
Treatment with lapatinib sequesters BRCA1 away from its nuclear repair substrates to the cytosol in triple negative breast cancer cells. (A–C) Cells were subjected to 1 µM lapatinib treatment for 16 hours and BRCA1 location was assessed by subcellular fractionation. Lapatinib induced cytosolic translocation of BRCA1 in (A) MDA-MB-231, (B) MDA-MB-453 and (C) MDA-MB-468 cells. Histone H1 and α-tubulin were used to test the purity of the nuclear and cytosolic fractions respectively. Quantification of BRCA1 levels was performed via densitometry. Shown is the representative data of three independent experiments (mean +/− SEM, **p<0.01).

We were interested in further deciphering the mechanism of contextual synthetic lethality between combined EGFR and PARP inhibitors. One mechanism by which the function of DNA repair proteins can be regulated is through protein-protein interactions and/or protein shuttling. We hypothesized that lapatinib-mediated reduction in DNA repair and subsequent persistence of DNA damage may be due to such regulation of key DNA repair proteins with EGFR. One of the major DNA repair proteins involved in HR-mediated DNA double strand break repair is BRCA1. Nuclear BRCA1 promotes HR-mediated DNA repair while cytosolic BRCA1 promotes apoptosis [14], [15]. Interestingly, as shown in Figure 4, the subcellular localization of BRCA1, which is predominantly located in the nucleus basally, was approximately two-fold reduced in the nucleus following lapatinib treatment. This coincides with a concomitant increase in cytosolic BRCA1 in MDA-MB-231 (Figure 4A), MDA-MB-453 (Figure 4B), and MDA-MB-468 (Figure 4C) cell lines.

**Figure 5:**
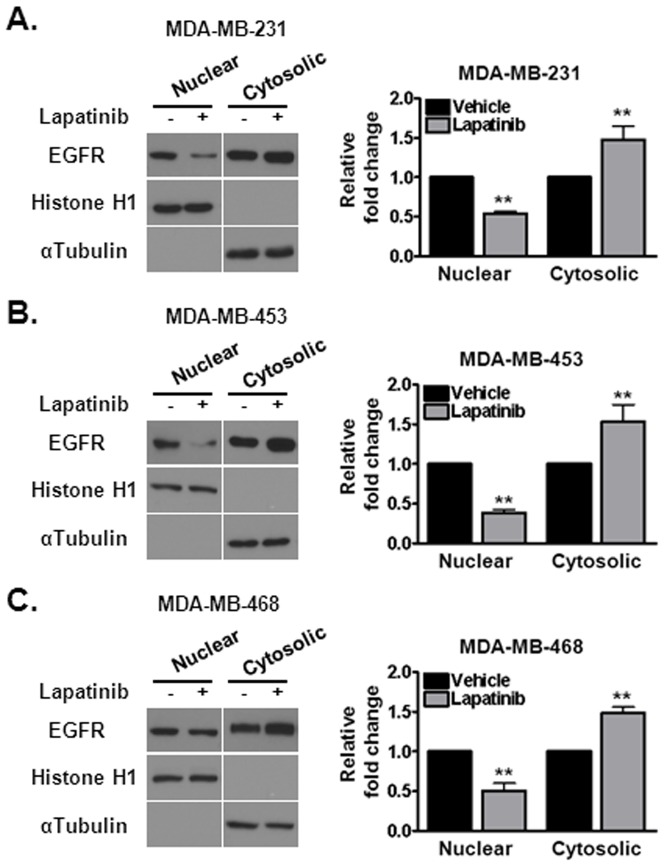
Treatment with lapatinib sequesters EGFR to the cytosol in triple negative breast cancer cells. (A–C) Cells were subjected to 1 µM lapatinib treatment for 16 hours and EGFR location was assessed by subcellular fractionation. Lapatinib induced cytosolic translocation of EGFR in (A) MDA-MB-231, (B) MDA-MB-453 and (C) MDA-MB-468 cells. Histone H1 and α-tubulin were used to test the purity of the nuclear and cytosolic fractions respectively. Quantification of EGFR levels was performed via densitometry. Shown is the representative data of three independent experiments (mean +/− SEM, **p<0.01).

Another important protein involved in DNA repair is EGFR itself. Upon DNA damage, EGFR translocates to the nucleus and promotes DNA repair. Since lapatinib inhibits EGFR, we hypothesized that reduction in DNA repair by lapatinib may also be due to sequestration of EGFR in the cytosol. Consistent with our hypothesis, the level of nuclear EGFR is reduced approximately two-fold following treatment with lapatinib in MDA-MB-231 (Figure 5A), MDA-MB-453 (Figure 5B), and MDA-MB-468 (Figure 5C) cell lines. Thus, the attenuation of DNA repair following lapatinib is due to, in part, sequestration of DNA double strand break repair proteins to the cytosol, away from their DNA repair substrates in the nucleus.

### Lapatinib attenuates the interaction between EGFR and BRCA1 in breast cancer cells

**Figure 6:**
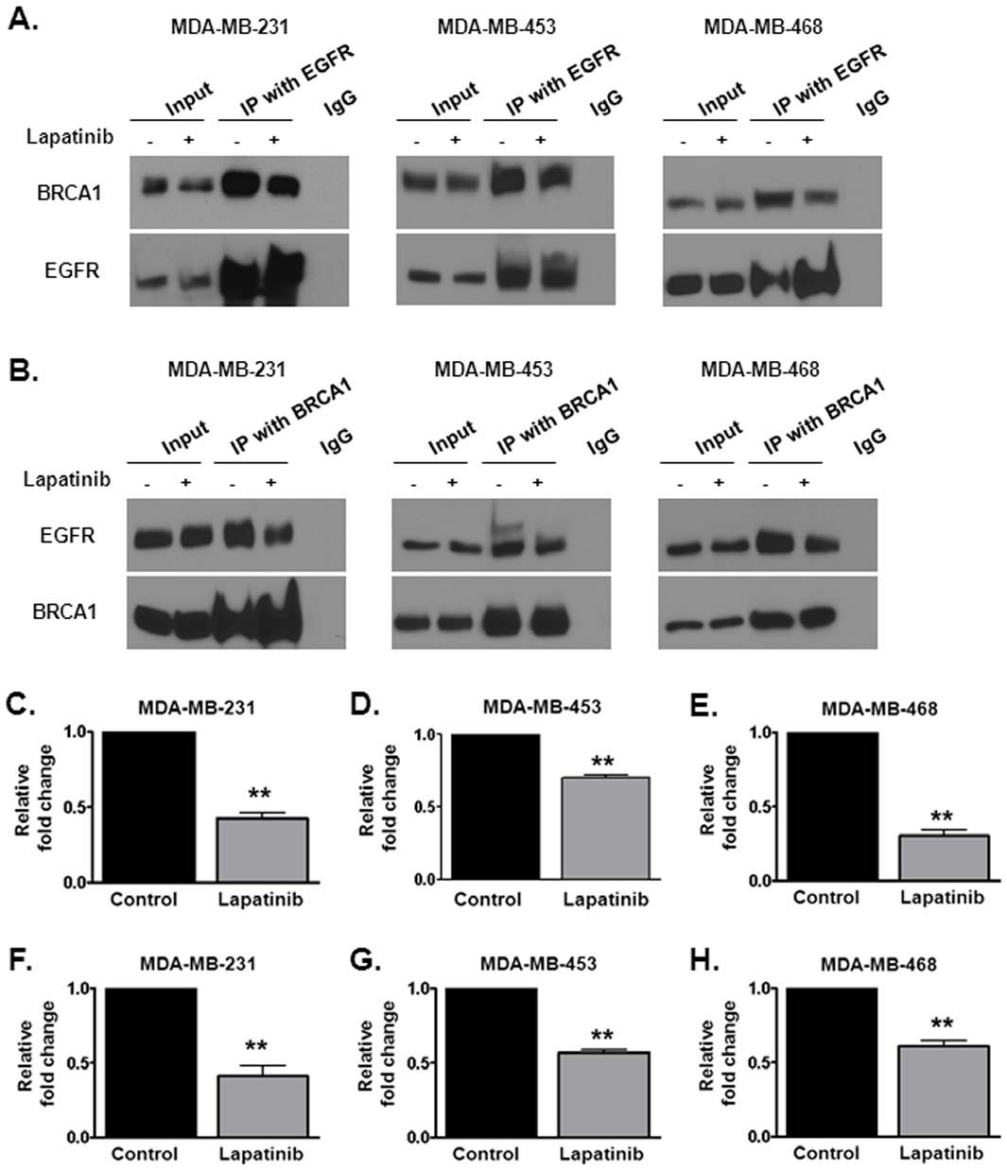
Lapatinib interrupts the interaction between BRCA1 and EGFR in triple negative breast cancer cell lines. (A–B) Reciprocal immunoprecipitation with (A) EGFR and (B) BRCA1 was performed in (from left to right) MDA-MB-231, MDA-MB-453 and MDA-MB-468 cells following 16 hours of vehicle or 1 µM lapatinib treatment. The levels of BRCA1 and EGFR in immunocomplexes were normalized to the amount of the reciprocal protein that was pulled down. (C–H) Quantification of EGFR and BRCA1 levels was performed via densitometry. Shown is the representative data of three independent experiments (mean +/− SEM, **p<0.01).

Since both EGFR and BRCA1 were both sequestered away from the nucleus by lapatinib we were interested in determining whether these two proteins interacted to regulate DNA repair. To determine the association of EGFR and BRCA1, we performed co-immunoprecipitation experiments in the TNBC cell lines MDA-MB-231, MDA-MB-453 and MDA-MB-468 (Figure 6A and 6B). As shown in Figure 6, EGFR and BRCA1 indeed can be found in the same immuno complex. Interestingly, a 35–70% reduction in BRCA1 levels was observed in the EGFR-immunocomplexes of cells treated with lapatinib (Figure 6A and 6C–6E). Similarly, EGFR and BRCA1 were again found together in a reciprocal immunoprecipitation. A 40–60% reduction in EGFR level was also observed in the BRCA1-immunocomplexes pulled down in cells treated with lapatinib (Figure 6B and 6F–6H). Thus, interruption of the BRCA1-EGFR complex may be one possible mechanism by which lapatinib attenuates DNA repair in breast cancer cells.

### Combination of Lapatinib and ABT-888 delays growth of triple negative breast cancer tumors in vivo

**Figure 7:**
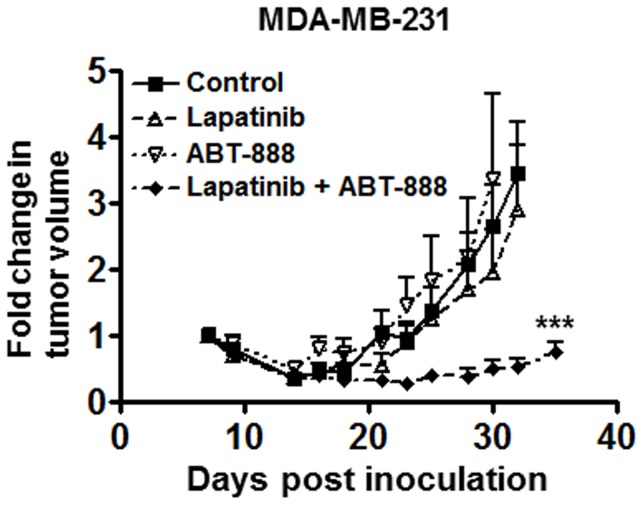
Combined EGFR and PARP inhibition delays the growth of orthotopic breast tumor xenografts in mice. MDA-MB-231 cells were injected into the mammary fat pads of mice. Once tumors were palpable, mice were treated with vehicle, 30 mg/kg/day lapatinib (b.i.d.), 100 mg/kg/day ABT-888 (b.i.d.), or combination of lapatinib and ABT-888. Treatment period was for 26 days and tumors were measured via caliper three times per week (n = 7, mean +/− SEM, ***p<0.001).

To validate our results *in vivo*, we assessed tumor growth delay in mice bearing orthotopic xenografts of MDA-MB-231 breast cancer cells. As shown in Figure 7 and similar to previous reports, administration of ABT-888 or lapatinib alone did not significantly delay tumor growth of MDA-MB-231 xenografts. However, the combination of lapatinib and ABT-888 significantly delayed tumor growth of these xenografts (>3 fold tumor growth delay in combination treatment vs. control, p<0.001). Thus, these results validated the synthetic lethal interactions between EGFR and PARP inhibition in triple negative breast tumors.

## Discussion

In this study, we report a contextual synthetic lethality with combined EGFR and PARP inhibition with lapatinib and ABT-888, respectively due to a transient DNA double strand break repair deficit induced by lapatinib and subsequent activation of the intrinsic pathway of apoptosis. Importantly, these results were validated *in vivo*. Interestingly, our data also suggest a novel regulation of HR-mediated repair involving EGFR and BRCA1 interaction and subcellular localization.

BRCA1 is a key nuclear shuttling protein which is essential in maintaining genomic stability and controlling cellular response to genotoxic stress. Sequestration of BRCA1 away from the nucleus may switch BRCA1 function from high fidelity DNA repair in the nucleus to activation of cell death signals in the cytoplasm [14], [15], [23], [24]. Thus, in addition to an induced DNA repair deficit, other potential mechanisms may explain the enhanced cytotoxicity to PARP inhibition following lapatinib-mediated BRCA1 nuclear export. For instance, the potential role of cytosolic BRCA1 in augmenting cell death pathways due to interaction with pro-apoptotic proteins or sequestration to mitochrondrial endomembranes may also explain the dramatic sensitivity of cells to combined EGFR and PARP inhibition [14], [15], [24], [25].

EGFR plays an essential role in carcinogenesis by modulating proliferation, differentiation, and the DNA damage response [10], [11], [26], [27], [28], [29], [30]. In particular, overexpression and amplification of the EGFR is present in the majority of TNBC as well as other cancers and portends poor prognosis, inferior survival, radioresistance, and treatment failures [28], [31], [32], [33]. Thus, this therapeutic approach may not only be feasible for TNBC, but other EGFR dysregulated tumors such as brain, lung, head and neck, and pancreas.

Interestingly, in all three models of triple negative breast cancer used in this study, a similar level of cytotoxicity was observed despite differential EGFR expression. Similar to previous reports, EGFR expression from low to high is as follows: MDA-MB-453, MDA-MB-231, and MDA-MB-468 (data not shown, [34]). Given the minor response of these cells to EGFR inhibition alone, this result is not unexpected. Additionally, the synthetic lethality may be due to the important role of EGFR in DNA repair. Following DNA damage EGFR binds to, among others, the catalytic subunit of DNA PK, a protein involved in non-homologous end joining (NHEJ) mediated DNA double strand break repair [35]. Additionally, activation of EGFR enhances double stranded break repair irrespective of p53 and KRAS mutational status [36]. We and others have recently shown that EGFR modulates HR-mediated double strand break repair as well [11], [36]. Our study suggests that similar to EGFR-mediated regulation of NHEJ, EGFR may regulate HR-mediated repair by interacting with BRCA1.

We also report for the first time to our knowledge that EGFR and BRCA1 can be found in the same protein complex, which is reduced by lapatinib. It is likely that these critical proteins involved in DNA damage response interact in the nucleus to augment DNA repair and since lapatinib sequesters both EGFR and BRCA1 to the cytosol, the interaction is abrogated following EGFR inhibition. It is interesting to speculate that other cofactors present in the nucleus are required to facilitate this interaction. We are actively investigating this possibility. Alternatively, the reduction of EGFR-BRCA1 interaction may relate to the role of BRCA1 in activation of apoptosis in the cytosol as mentioned above. Perhaps, the disruption of EGFR-BRCA1 interaction may allow BRCA1 to dissociate from the DNA repair complex and translocate to the cytosol to activate apoptosis.

Recent reports suggest that triple negative breast cancer is a heterogeneous group of tumors, with variation in morphology, mutations, and signaling which inevitably lead to differences in tumor biology and treatment response. In one study, it was suggested that there are six distinct groups of triple negative breast cancers based on gene expression profiles: basal-like 1, basal-like 2, immunomodulatory, mesenchymal, mesenchymal stem-like, and luminal androgen receptor [37]. In this study, the cell lines used represent three of the sub-types, MDA-MB-468 (basal-like 1), MDA-MB-231 (mesenchymal stem-like), and MDA-MB-453 (luminal androgen receptor). Interestingly, the differential gene expression profiles of the 3 subtypes suggest rationale for such a synthetic lethal response from combination EGFR/PARP inhibition. The basal-like 1 tumors have elevated expression of DNA damage response genes, while mesenchymal tumors were enriched for genes implicated in growth factor signaling pathways, including EGFR. The luminal androgen receptor subtype (MDA-MB-453) rely heavily on hormonally regulated pathways, but have *PIK3CA* and *PTEN* mutations, which have been implicated in altered DNA repair responses. The other 2 subtypes, immunomodulatory and mesenchymal, may not exhibit sensitivity to this combination based on gene profiles involving immune system and cell motility, respectively. However, because the majority of triple negative breast cancer is basal-like, our results suggest that combination EGFR and PARP inhibition may potentially impact a large portion of the triple negative breast cancer patient population.

In summary our intriguing and novel results point to the potential broader utility of PARP inhibitors in breast cancer beyond hereditary BRCA1-and BRCA2-deficient tumors by combining it with EGFR inhibitors such as lapatinib. Moreover, the discovery of the novel EGFR-BRCA1 interaction may lead to other therapeutic targets for the highly aggressive TNBC.

## Supporting Information

Table S1 **Lapatinib treatment does not induce significant changes in cell cycle distribution.** MDA-MB-231, MDA-MB-453 and MDA-MB-468 breast cancer cell lines were seeded for cell cycle analysis and exposed to 1 µM lapatinib or vehicle treatment. Cell cycle distribution was analyzed 16 and 24 hours following lapatinib treatment. Experiment was performed in triplicate and shown is the mean percentage of cells ± SEM.
(DOCX)

Materials and Methods S1 (DOCX)
