# Supplementary material for: Synthetic Lethal Interactions between EGFR and PARP Inhibition in Human Triple Negative Breast Cancer Cells
Source: PLoS One. 2012 Oct 11;7(10):e46614. doi: 10.1371/journal.pone.0046614 (PMC3469581; doi:10.1371/journal.pone.0046614)
Supplement: Table S1 — Lapatinib treatment does not induce significant changes in cell cycle distribution. MDA-MB-231, MDA-MB-453 and MDA-MB-468 breast cancer cell lines were seeded for cell cycle analysis and exposed to 1 µM lapatinib or vehicle treatment. Cell cycle distribution was analyzed 16 and 24 hours following lapatinib treatment. Experiment was performed in triplicate and shown is the mean percentage of cells ± SEM. (DOCX) [file pone.0046614.s001.docx]

**Table S1:** Lapatinib treatment does not induce significant changes in cell cycle distribution.

| Cell line | Time (hours) | Treatment | %G1 ± SEM | %S ± SEM | %G2/M ± SEM |
| --- | --- | --- | --- | --- | --- |
| MDA-MB-231 | 16 | Vehicle | 47.22 ± 0.22 | 26.28 **±** 1.91 | 26.50 **±** 2.13 |
|  | 16 | Lapatinib | 50.98 ± 4.88 | 23.66 **±** 2.77 | 25.36 **±** 2.11 |
|  | 24 | Vehicle | 47.72 ± 2.64 | 27.56 **±** 2.10 | 24.72 **±** 0.54 |
|  | 24 | Lapatinib | 52.34 ± 1.36 | 25.13 **±** 0.16 | 22.53 **±** 1.52 |
| MDA-MB-453 | 16 | Vehicle | 40.16 ± 3.15 | 29.06 ± 1.96 | 30.77 ± 1.19 |
|  | 16 | Lapatinib | 44.63 ± 2.56 | 24.88 ± 4.00 | 27.72 ± 3.78 |
|  | 24 | Vehicle | 44.12 ± 1.77 | 23.43 ± 1.95 | 32.45 ± 0.17 |
|  | 24 | Lapatinib | 48.24 ± 4.12 | 22.50 ± 1.93 | 28.26 ± 2.19 |
| MDA-MB-468 | 16 | Vehicle | 45.46 ± 0.33 | 18.45 ± 2.15 | 36.08 ± 2.49 |
|  | 16 | Lapatinib | 48.92 ± 3.95 | 18.37 ± 1.80 | 32.7 ± 2.15 |
|  | 24 | Vehicle | 46.49 ± 2.19 | 22.07 ± 1.37 | 31.44 ± 0.81 |
|  | 24 | Lapatinib | 49.5 ± 0.69 | 20.83 ± 0.26 | 29.67 ± 0.95 |
