# Supplementary material for: Synthetic Lethal Interactions between EGFR and PARP Inhibition in Human Triple Negative Breast Cancer Cells
Source: PLoS One. 2012 Oct 11;7(10):e46614. doi: 10.1371/journal.pone.0046614 (PMC3469581; doi:10.1371/journal.pone.0046614)
Supplement: Materials and Methods S1 — (DOCX) [file pone.0046614.s002.docx]

**Supplemental Materials and Methods**

*Cell cycle analysis*

Cell cycle distribution was measured as previously described [1]. 8x10^4^ cells were seeded in 100 mm^2^ dishes and treated with 1μM lapatinib or vehicle. Cells were collected and fixed 16 and 24 hours following lapatinib treatment, treated with RNAse (Sigma, catalog # R-4875), stained with propidium iodide (PI), and read on FACSCalibur using CellQuest. Data was analyzed using ModFit LT by Verity Software Inc.

**Reference**

1. Nowsheen S, Bonner JA, LoBuglio AF, Trummell H, Whitley AC, et al. (2011) Cetuximab Augments Cytotoxicity with Poly (ADP-Ribose) Polymerase Inhibition in Head and Neck Cancer. PLoS ONE 6: e24148.
